# Supplementary material for: Social information use shapes the coevolution of sociality and virulence
Source: Evolution. 2022 Apr 23;76(6):1153–69. doi: 10.1111/evo.14491 (PMC9322624; doi:10.1111/evo.14491)
Supplement: Supplementary file 1 — Supporting Information [file EVO-76-1153-s001.pdf]

SUPPLEMENTARY MATERIAL:
SOCIAL INFORMATION USE SHAPES THE COEVOLUTION OF SOCIALITY AND VIRULENCE

Ben Ashby[1,2*] and Damien R Farine[3,4,5]

1. Department of Mathematical Sciences, University of Bath, Bath, BA2 7AY, UK
2. Department of Mathematics, Simon Fraser University, Burnaby, BC, V5A 1S6, Canada
3. Department of Evolutionary Biology and Environmental Studies, University of Zurich, 8047 Zurich, Switzerland
4. Max Planck Institute of Animal Behavior, 78315 Radolfzell, Germany
5. Centre for the Advanced Study of Collective Behaviour, University of Konstanz, 78464 Konstanz, Germany

A. FULL MODEL

A.1: BASIC REPRODUCTIVE RATIO: SOCIAL INFORMATION

To determine the viability of social information transmission, consider the dynamics when social information is rare:

$$\frac{dS_G}{dt} = \frac{E^2}{N}\left(\tau S_P(S_G + I_G) - \beta S_G I_P\right) - adS_G + \gamma I_G - \sigma S_G \tag{S1a}$$

$$\frac{dI_G}{dt} = \frac{E^2}{N}\left(\beta S_G I_P + \tau I_P(S_G + I_G)\right) - (ad + \alpha + \gamma + \sigma)I_G \tag{S1b}$$

One can use the next-generation method (Hurford et al. 2010) to calculate the basic reproductive ratio for social information, R_0^I, which first requires deriving the Jacobian of this system:

$$J = \begin{pmatrix} \frac{E^2}{N}(\tau S_P - \beta I_P) - ad - \sigma & \frac{E^2 \tau S_P}{N} + \gamma \\ \frac{E^2}{N} I_P(\beta + \tau) & \frac{E^2 \tau I_P}{N} - (ad + \alpha + \gamma + \sigma) \end{pmatrix} \tag{S2}$$

Next, one must separate the Jacobian into components, F and V such that $J = F - V$ with:

$$F = \frac{E^2 \tau}{N}\begin{pmatrix} S_P & S_P \\ I_P & I_P \end{pmatrix} \tag{S3}$$

$$V = \begin{pmatrix} \frac{E^2 \beta I_P}{N} + ad + \sigma & -\gamma \\ -\frac{E^2 \beta I_P}{N} & ad + \alpha + \gamma + \sigma \end{pmatrix} \tag{S4}$$

The next-generation matrix is given by $N_G = FV^{-1}$, and the basic reproductive ratio for social information is equal to the largest eigenvalue:

$$R_0^I = \frac{\tau E^2 \left(\Gamma_\gamma^I I_P + S_P(\Gamma_\alpha^I + \gamma)\right) N + \beta E^2 I_P (I_P + S_P)}{N\big((ad+\sigma)(\Gamma_\alpha^I + \gamma)N + \beta E^2 I_P \Gamma_\alpha^I\big)} \tag{S5}$$

where $\Gamma_\alpha^I = ad + \alpha + \sigma$ and $\Gamma_\gamma^I = ad + \gamma + \sigma$.

A.2: Basic reproductive ratio: disease

We now follow the same method described above, but for disease. First consider the dynamics when disease is rare:

$$\frac{dI_P}{dt} = \frac{E^2}{N}(\beta S_P (I_P + I_G) - \tau I_P S_G) - (d + \alpha + \gamma) I_P + \sigma I_G \tag{S6a}$$

$$\frac{dI_A}{dt} = \frac{E^2}{N}(\beta S_G (I_P + I_G) + \tau I_P S_G) - (ad + \alpha + \gamma + \sigma) I_G \tag{S6b}$$

The Jacobian of this system is:

$$J = \begin{pmatrix} \frac{E^2}{N}(\beta S_P - \tau S_G) - (d + \alpha + \gamma) & \frac{E^2 \beta S_P}{N} + \sigma \\ \frac{E^2}{N} S_G (\beta + \tau) & \frac{E^2 \beta S_G}{N} - (ad + \alpha + \gamma + \sigma) \end{pmatrix} \tag{S7}$$

Separating the Jacobian into components, F and V such that $J = F - V$:

$$F = \frac{E^2 \beta}{N} \begin{pmatrix} S_P & S_P \\ S_G & S_G \end{pmatrix} \tag{S8}$$

$$V = \begin{pmatrix} \frac{E^2 \tau S_G}{N} + d + \alpha + \gamma & -\sigma \\ -\frac{E^2 \tau S_G}{N} & ad + \alpha + \gamma + \sigma \end{pmatrix} \tag{S9}$$

The next-generation matrix is again given by $N_G = FV^{-1}$, and the basic reproductive ratio for disease is equal to the largest eigenvalue:

$$R_0^D = \frac{\beta E^2\big((\Gamma_D + \sigma)S_G + S_P(\Gamma_\alpha^I + \gamma)\big)N + \tau E^2 S_G(S_P + S_G))}{N(\Gamma_D(\Gamma_\alpha^I + \gamma)N + \tau E^2 S_G \Gamma_\alpha^I)} \tag{S10}$$

where $\Gamma_D = d + \alpha + \gamma$.

A.3: Host fitness

Consider the dynamics of a rare host mutant with contact effort $c_m \approx c$ into an established resident population at equilibrium (denoted by asterisks):

$$\frac{dS_P^m}{dt} = (b - qN^*)N_m - \frac{E_m E S_P^m}{N^*}\big(\beta(I_P^* + I_G^*) + \tau(S_G^* + I_G^*)\big) - dS_P^m + \gamma I_P^m + \sigma S_G^m \tag{S11a}$$

$$\frac{dS_G^m}{dt} = \frac{E_m E}{N^*}\big(\tau S_P^m(S_G^* + I_G^*) - \beta S_G^m(I_P^* + I_G^*)\big) - adS_G^m + \gamma I_G^m - \sigma S_G^m \tag{S11b}$$

$$\frac{dI_P^m}{dt} = \frac{E_m E}{N^*}\big(\beta S_P^m(I_P^* + I_G^*) - \tau I_P^m(S_G^* + I_G^*)\big) - (d + \alpha + \gamma)I_P^m + \sigma I_G^m \tag{S11c}$$

$$\frac{dI_G^m}{dt} = \frac{E_m E}{N^*}\big(\beta S_G^m(I_P^* + I_G^*) + \tau I_P^m(S_G^* + I_G^*)\big) - (ad + \alpha + \gamma + \sigma)I_G^m \tag{S11d}$$

The Jacobian of this system is:

$$J = \begin{pmatrix} b - qN^* - \frac{C_m}{N}(\beta I^* + \tau G^*) - d & b - qN^* + \sigma & b - qN^* + \gamma & b - qN^* \\ \frac{C_m}{N}\tau G^* & -\frac{C_m}{N}\beta I^* + ad + \sigma & 0 & \gamma \\ \frac{C_m}{N}\beta I^* & 0 & -\frac{C_m}{N}\tau G^* - (d + \alpha + \gamma) & \sigma \\ 0 & \frac{C_m}{N}\beta I^* & \frac{C_m}{N}\tau G^* & -(ad + \alpha + \gamma + \sigma) \end{pmatrix} \tag{S12}$$

where $C_m = E_m E$, $G^* = S_G^* + I_G^*$ and $I^* = I_P^* + I_G^*$ for notational convenience. Separating the Jacobian into components, F and V such that $J = F - V$:

$$F = \begin{pmatrix} b - qN^* & b - qN^* & b - qN^* & b - qN^* \\ 0 & 0 & 0 & 0 \\ 0 & 0 & 0 & 0 \\ 0 & 0 & 0 & 0 \end{pmatrix} \quad (S13)$$

$$V = \begin{pmatrix} \frac{C_m}{N}(\beta I^* + \tau G^*) + d & -\sigma & -\gamma & 0 \\ -\frac{C_m}{N}\tau G^* & \frac{C_m}{N}\beta I^* + ad + \sigma & 0 & -\gamma \\ -\frac{C_m}{N}\beta I^* & 0 & \frac{C_m}{N}\tau A^* + d + \alpha + \gamma & -\sigma \\ 0 & -\frac{C_m}{N}\beta I^* & -\frac{C_m}{N}\tau G^* & ad + \alpha + \gamma + \sigma \end{pmatrix} \quad (S14)$$

The next-generation matrix is given by $N_G = FV^{-1}$, and the invasion fitness, $w_H(E_m, E)$, is equal to the largest eigenvalue of this matrix, which we omit here as it is a lengthy expression that offers no analytical insights. The host fitness gradient is then given by $f_H(E) = \left.\frac{\partial w_H}{\partial E_m}\right|_{E_m=E}$, and singular strategies, E^*, occur when $f_H(E^*) = 0$. Again, we omit the fitness gradient here as it is a lengthy expression. The singular strategy is evolutionarily stable if $\left.\frac{\partial^2 w_H}{\partial E_m^2}\right|_{E_m=E=E^*} < 0$ (giving a continuously stable strategy), otherwise it is evolutionarily unstable.

B. FAST SOCIAL INFORMATION APPROXIMATION

B.1: BASIC REPRODUCTIVE RATIO: SOCIAL INFORMATION

In the fast social information approximation, the proportion of the population in the good information state G for a given value of E, G_E, rapidly reaches equilibrium. Letting $G_E = \frac{1}{N}(S_G + I_G)$, we see in the main text that:

$$\frac{dG_E}{dt} \approx (\tau E^2(1 - G_E) - \sigma)G_E = 0 \quad (S15)$$

Thus, either no individuals are in the good information state, or $G_E^* = 1 - \frac{\sigma}{\tau E^2}$ when $\sigma < \tau E^2$. The basic reproductive ratio for social information is therefore:

$$\tilde{R}_0^I = \frac{\tau E^2}{\sigma} \quad (S16)$$

as social information can only spread when $\tilde{R}_0^I > 1$.

B.2: BASIC REPRODUCTIVE RATIO: DISEASE

In the fast approximation model,

$$\frac{dI}{dt} = \frac{\beta E^2 SI}{N} - (d(1-(1-a)G_E^*) + \alpha + \gamma)I \tag{S17}$$

When disease is rare $(I \approx 0, S \approx N)$, we have:

$$\frac{1}{I}\frac{dI}{dt} \approx \beta E^2 - (d(1-(1-a)G_E^*) + \alpha + \gamma) \tag{S18}$$

Rearranging $\frac{1}{I}\frac{dI}{dt} > 0$, we see that the basic reproductive ratio for disease is:

$$\tilde{R}_0^D = \frac{\beta E^2}{d(1-(1-a)G_E^*) + \alpha + \gamma} \tag{S19}$$

B.3: HOST FITNESS

We can approximate the invasion dynamics of a rare host mutant when social information dynamics are fast by:

$$\frac{dS_m}{dt} = (b - qN^*)N_m - \frac{\beta E_m E S_m I^*}{N^*} - d(1-(1-a)G_E^m)S_m + \gamma I_m \tag{S20a}$$

$$\frac{dI_m}{dt} = \frac{\beta E_m E S_m I^*}{N^*} - (d(1-(1-a)G_E^m) + \alpha + \gamma)I_m \tag{S20b}$$

where $G_E^m = \max\left(\frac{E_m(\tau E^2 - \sigma)}{E^2 E_m \tau + \sigma(E - E_m)}, 0\right)$. The Jacobian of this system is:

$$J = \begin{pmatrix} b - qN^* - \frac{\beta E_m E I^*}{N^*} - d(1-(1-a)G_E^m) & b - qN^* + \gamma \\ \frac{\beta E_m E I^*}{N^*} & -(d(1-(1-a)G_E^m) + \alpha + \gamma) \end{pmatrix} \tag{S21}$$

Separating the Jacobian into components, F and V such that $J = F - V$:

$$F = \begin{pmatrix} b - qN^* & b - qN^* \\ 0 & 0 \end{pmatrix} \quad (S22)$$

$$V = \begin{pmatrix} \frac{\beta E_m E I^*}{N^*} + d(1-(1-a)G_E^m) & -\gamma \\ -\frac{\beta E_m E I^*}{N^*} & d(1-(1-a)G_E^m) + \alpha + \gamma \end{pmatrix} \quad (S23)$$

The next-generation matrix is again given by $N_G = FV^{-1}$, and the invasion fitness, $w_H(E_m, E)$, is equal to the largest eigenvalue of this matrix, which we omit here as it is a lengthy expression which offers no analytical insights. The process for finding the fitness gradient, singular strategies, and evolutionary stability are as described above.

B.4: Parasite fitness

In the fast social information approximation, the invasion dynamics of the rare parasite mutant (M) with traits α_M and $\beta(\alpha_M)$, are given by:

$$\frac{dI_M}{dt} = \frac{\beta(\alpha_M)E^2 S^* I_M}{N^*} - (d(1-(1-a)G_E^*) + \alpha_M + \gamma)I_M \quad (S24)$$

Parasite invasion fitness is then simply equal to the per-capita growth rate when rare, $w_P(\alpha_M, \alpha) = \frac{1}{I_M}\frac{dI_M}{dt}$. The fitness gradient, $f_P(\alpha) = \left.\frac{\partial w_p}{\partial \alpha_M}\right|_{\alpha_M = \alpha}$, is then:

$$f_P(\alpha) = \frac{d\beta}{d\alpha}\left(\frac{\tau E^2(ad + \alpha + \gamma) + d\sigma(1-a)}{\beta(\alpha)\tau E^2}\right) - 1 \quad (S25)$$

A singular strategy, α^*, occurs when $f_P(\alpha^*) = 0$, which requires:

$$\frac{d\beta}{d\alpha} = \frac{\beta(\alpha)\tau E^2}{\tau E^2(ad + \alpha + \gamma) + d\sigma(1-a)} \quad (S26)$$

Now consider the basic reproductive ratio from equation (S19), with $\beta = \beta(\alpha)$:

$$\tilde{R}_0^D(\alpha) = \frac{\beta(\alpha)E^2}{d(1-(1-a)G_E^*) + \alpha + \gamma} \quad (S27)$$

where $G_E^* = 1 - \frac{\sigma}{\tau E^2}$ when $\sigma < \tau E^2$ and is 0 otherwise. Taking the derivative of $\tilde{R}_0^D(\alpha)$, setting this to 0 and rearranging, we find that:

$$\frac{d\beta}{d\alpha} = \frac{\beta(\alpha)\tau E^2}{\tau E^2(ad + \alpha + \gamma) + d\sigma(1 - a)} \qquad (S28)$$

which is identical to equation (S27). Hence, evolution will maximise $\tilde{R}_0^D(\alpha)$.

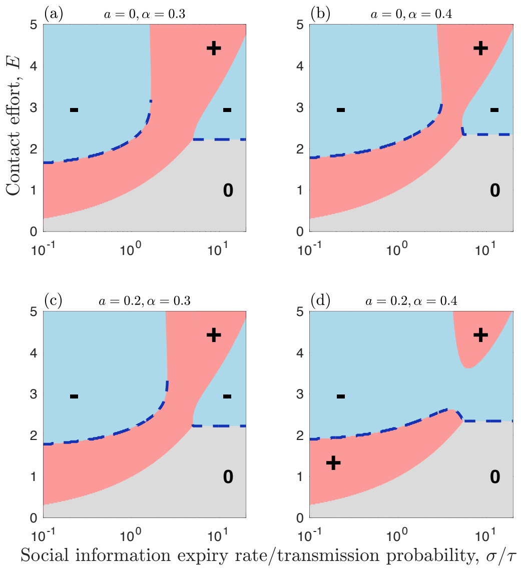

Figure S1. The evolution of sociality arising from social information and disease transmission (fast information approximation). Evolution of contact effort, E, as a function of the social information expiry rate divided by the transmission probability, σ/τ, and for different values of the benefits of social information, a, and costs of disease, α. Pink (+) and blue (-) regions indicate when the fitness gradient is positive (E will increase) and negative (E will decrease), respectively. Grey (0) regions indicate when there is no selection as social information and disease are both absent from the population. Blue dashed curves indicate stable or semi-stable levels of contact effort. Fixed parameters as in Fig. 2, except $\beta = 0.2$.

REFERENCES

Hurford, A., D. Cownden, and T. Day. 2010. Next-generation tools for evolutionary invasion analyses. J. R. Soc. Interface 7:561–571.
